# Health Behavior and Cancer Prevention among Adults with Li-Fraumeni Syndrome and Relatives in Germany—A Cohort Description

**DOI:** 10.3390/curroncol29100614

**Published:** 2022-10-15

**Authors:** Juliane Nees, Senta Kiermeier, Farina Struewe, Myriam Keymling, Imad Maatouk, Christian P. Kratz, Sarah Schott

**Affiliations:** 1 Department of Gynecology and Obstetrics, University Hospital Heidelberg, Im Neuenheimer Feld 440, 69120 Heidelberg, Germany; 2 Section of Psychosomatic Medicine, Psychotherapy and Psychooncology, Department of Internal Medicine II, Julius-Maximilian University Würzburg, Oberdürrbacher Straße 6, 97080 Würzburg, Germany; 3 Department of Pediatric Hematology and Oncology, Hannover Medical School, Carl-Neuberg-Str. 1, 30625 Hanover, Germany; 4 German Cancer Research Center (DKFZ), Department of Radiology, Im Neuenheimer Feld 280, 69120 Heidelberg, Germany

**Keywords:** pathogenic *TP53* germline variant, Li-Fraumeni syndrome, cancer prevention, physical activity, cancer predisposition, SF-12, MEDAS

## Abstract

Li-Fraumeni-syndrome (LFS) is a rare, highly penetrant cancer predisposition syndrome (CPS) caused by pathogenic variants (PVs) in *TP53*. Physical activity (PA) and a Mediterranean diet lead to cancer reduction or survival benefits and increased quality of life (QoL), but this is yet unstudied among LFS. *TP53* PV carriers (PVC) and their relatives were questioned on dietary patterns (Mediterranean Diet Adherence Screener), PA (Freiburg Questionnaire), QoL (Short-form-Health-Survey-12), smoking, alcohol consumption and perception of cancer risk in a German bi-centric study from March 2020–June 2021. The study enrolled 70 PVC and 43 relatives. Women compared to men (6.49 vs. 5.38, *p* = 0.005) and PVC to relatives (6.59 vs. 5.51; *p* = 0.006) showed a healthier diet, associated with participation in surveillance (*p* = 0.04) and education (diet *p* = 0.02 smoking *p* = 0.0003). Women smoked less (2.91 vs. 5.91 packyears; *p* = 0.03), psychological well-being was higher among men (SF-12: males 48.06 vs. females 41.94; *p* = 0.004). PVC rated their own cancer risk statistically higher than relatives (72% vs. 38%, *p* < 0.001) however, cancer risk of the general population was rated lower (38% vs. 70%, *p* < 0.001). A relative’s cancer-related death increased the estimated personal cancer risk (*p* = 0.01). The possibilities of reducing cancer through self-determined health behavior among PVC and relatives has not yet been exhausted. Educating families with a CPS on cancer-preventive behavior requires further investigation with regard to acceptance and real-life implementation.

## 1. Introduction

Li-Fraumeni syndrome (LFS) is a rare and highly penetrant cancer predisposition syndrome (CPS) caused by pathogenic/likely pathogenic variants (PVs) in the *TP53* tumor suppressor gene. Complex surveillance strategies are recommended for early cancer detection and increased overall survival [1,2]. Individuals and relatives are known to carry an emotional burden [3]. A recent study has outlined the complexity of LFS implementing a new definition of the LFS spectrum rather than one syndrome, aiming at individualizing cancer prevention and surveillance strategies *TP53* PV carriers [4].

Lifestyle factors such as obesity, physical activity, diet, alcohol, and smoking are considered modifiable cancer risk factors [5,6]. According to the German Robert Koch Institute, a healthy diet, physical activity, and body weight have a high prevention potential for cancer [7]. In cancer patients, a survival benefit and an increased quality of life (QoL) through physical activity (PA) is known throughout therapy and beyond [8,9,10,11]. PA was further found to have an attributed risk reduction of up to 20% for breast cancer (BC) in cases of familiar risk and genetic susceptibility [12]. Dietary studies focusing on the Mediterranean diet have shown a protective anticancer effect in the general population [13,14]. A healthy diet reduced the risk of developing BC for women with a germline *BRCA1/2* PV [15]. Beside PA and dietary patterns, lifestyle changes in terms of avoidance of well-recognized carcinogens such as smoking and alcohol consumption [16] might be preventive options, especially for people with a CPS who need health education regarding a cancer preventive lifestyle. Altogether, there are several options for self-determined cancer prevention strategies, yet not established within German guidelines.

The purpose of this study was to analyze lifestyle factors including nutrition, PA, and smoking habits among *TP53* PV carriers and their social environment. To the best of our knowledge, this is the first study to address these questions.

## 2. Methods

### 2.1. Study Population

This questionnaire-based bicentric study conducted at the University Hospitals in Heidelberg and Hannover, Germany, was open to *TP53* PV carriers ≥18 years and their relatives between March 2020–June 2021. All *TP53* PV carriers and relatives consulting these centers were offered study participation independent of their current heath or treatment status (active recruitment). In addition, the study was promoted through patient organizations, social media channels, newsletters, and personal contacts. Adequate German language skills were a requirement for participation. All participants provided written informed consent. Results were correlated with *TP53* PV carriers vs. relative status, sex, income, and body weight.

### 2.2. Measurements

Sociodemographic data were assessed using 15 self-designed items (Supplementary Material File S1). Nutrition habits were evaluated employing the validated German version of the Mediterranean Diet Adherence Screener (MEDAS) instrument with 12 items on food intake and analyzed according to the published manual with a maximum of 14 points [17,18]. Physical activity (PA) was measured using the validated 12-item Freiburg questionnaire, covering PA at work, during leisure time, and sports. The claimed duration of activities was converted to metabolic equivalents (MET) as previously described [19]. The German version of the well-known Short Form Health Survey 12 (SF-12 Version 2.0,Hogrefe, Göttingen, Germany ) was used for health-related QoL measures, subdivided into mental and physical health [20]. In addition, all study participants were asked to estimate their own cancer risk and the risk of the general population; relatives were asked about the absolute cancer risk of *TP53* PV carriers. Part of the questionnaire can be found as Additional File S1.

### 2.3. Statistical Analysis

The statistical analysis was performed using R 4.1.1. (Vienna, Austria) Empirical distributions were determined by absolute and relative frequencies for categorical variables using available case analysis. For continuous variables and composite scores, the mean value, standard deviation, minimum, maximum, and median were calculated. For group comparison of numerical data, we performed Mann–Whitney U tests as the presumptions for an ANOVA were not met. Associations were determined using a correlation test based on the Pearson’s coefficient for continuous variables and the Spearman’s coefficient for ordinal variables. *p*-values ≤ 0.05 were regarded as significant.

## 3. Results

### 3.1. Study Cohort, Cancer Experiences

**Figure 1:**
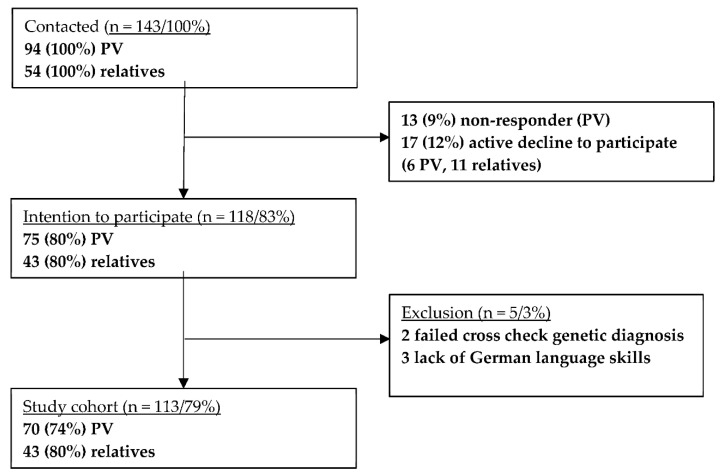
Flow chart of active recruitment. PV = pathogenic/likely pathogenic *TP53* variant carriers.

The study enrolled 113 participants (81 women, 72%; 32 men, 28%): 70 *TP53* PV carriers (58 women, 83%; 12 men, 17%) and 43 relatives (23 women, 53%; 20 men, 47%). The recruitment flow chart is shown in Figure 1. The social environment group comprised 20 spouses, 4 partners, 19 family members such as adult children, parents or siblings.

The mean time since diagnosis of the *TP53* PV was 4 years (range 0–25 years). For 3% of the study population, the diagnosis was made only in the past year, whereas 4% had been living with the diagnosis for more than 10 years.

Sociodemographic data are shown in Table 1. In brief, the average participant was aged 40 years (mean, range 20–69 years), married (*n* = 65, 58%), and had children (n = 68, 60%). A statutory health insurance (*n* = 105, 93%) was most common. More than half of the participants were currently employed (*n* = 66, 58%), with an average monthly net income of EUR 3,380, and 38% had a university degree (*n* = 42).

Regarding the prevalence of cancer, 51 study participants (45%) had received at least one cancer diagnosis (*TP53* PV carriers: *n* = 47, 67%; 4 relatives: *n* = 4, 9%). 4% (*n* = 4) of the study population reported losing a sibling or child to cancer. Their own risk of developing cancer was rated significantly higher by *TP53* PV carriers than by their relatives (72% vs. 38%, *p* < 0.001). In contrast, the risk of the general population to develop cancer was estimated statistically significantly lower than by the relatives (38% vs. 70% *p* < 0.001). The relatives estimated the mean absolute cancer risk for the *TP53* PV carriers as 80%. A death of a relative from cancer was the only factor with a statistically significant impact on the estimation of the *TP53* PV carriers’ own cancer risk (*p* = 0.01), but not among relatives (*p* = 0.33). In contrast, neither a personal cancer diagnosis nor that of a family member influenced the perceived cancer risk estimate (carriers: *p* = 0.89; *p* = 0.30; relatives: *p* = 0.12; *p* = 0.69). In our study cohort, 80% of *TP53* PV carriers followed at least some of the recommended surveillance recommendations [1,2].

### 3.2. Lifestyle and Dietary Habits, Physical Activity, and Physical Wellbeing

The average participant was pre-obese with a mean BMI of 26 kg/m^2^ (range 18–66) without any significant gender imbalance (see Table 2). The groups were equally distributed for BMI with almost half (51% *TP53* PV carriers and 52% relatives) being normal or underweighted (BMI < 25 kg/m^2^), followed by a third meeting the criteria of pre-obesity (29% and 31% for BMI 25–<30 kg/m^2^), 12% and 10% for obesity class I (BMI 30–<35 kg/m^2^), 6% and 2% for class II (BMI 35–<40 kg/m^2^), 3% and 5% for class III (BMI > 40 kg/m^2^), respectively. In summary, 20% of the study population was considered obese (BMI ≥ 30 kg/m^2^).

The entire study population tended not to follow a Mediterranean diet, shown by an overall MEDAS score of 6. The following statistically significant differences were found: (1) Women had healthier diets than men (6.49 vs. 5.28, *p* = 0.005); and (2) *TP53* PV carriers than their relatives (6.59 vs. 5.51; *p* = 0.006).

Regarding smoking behavior, it was found that women smoked less often than men (2.91 vs. 5.91 packyears) (*p* = 0.03), and *TP53* PV carriers tended to smoke less than their relatives (1.93 vs. 4.51 packyears, *p* = 0.14). Overall, the proportion of smokers in the entire study population was high with 73.5% (77.1% *TP53* PV carriers vs. 67.4% relatives). Alcohol consumption was almost the same in the entire study population without significant gender differences (*p* = 0.21) or *TP53* PV carriers versus relative status (*p* = 0.64).

PA as assessed with the Freiburg questionnaire revealed men to be slightly more physically active than women (21.82 vs. 21.02, *p* = 0.48) and *TP53* PV carriers more active than their relatives (22.63 vs. 18.99), without statistically significant differences (*p* = 0.28). Regarding physical and psychological well-being (SF-12), the entire study population rated their physical well-being nearly the same as the average population (48.83), while the psychological well-being appeared to be lower (43.70) than average. Men felt physically (51.19 vs. 47.88, *p* = 0.17) and psychologically (48.06 vs. 41.94, *p* = 0.004) statistically significantly better than women. The relatives of LFS carriers showed a trend toward lower physical well-being (men: 50.49 vs. 52.35; women 47.37 vs. 48.09, *p* = 0.65) but higher psychological well-being than LFS carriers (45.99 vs. 42.28, *p* = 0.07). The only statistically significant difference among PV carriers with respect to a personal cancer history was the physical well-being (53.46 vs. 46.51, *p* = 0.018) which was better among former cancer patients, whereas differences in the areas PA (*p* = 0.69), diet (*p* = 0.86), and alcohol consumption (*p* = 0.48) were not statistically significant. The cohort size with data on smoking behavior was too small for analysis.

In a further step, we examined factors that correlate with lifestyle habits: A high BMI (>25 kg/m²) was not associated with unhealthy behavior such as smoking or alcohol consumption. There was even a trend of more alcohol consumption among thinner participants (*p* = 0.14) and a significant difference toward more alcohol intake among those with higher incomes (*p* = 0.04). The level of education correlated significantly with healthy behaviors in relation to diet (*p* = 0.02) and less smoking (*p* = 0.0003), but not with PA (*p* = 0.39) or alcohol consumption (*p* = 0.81). Losing a close person due to cancer was associated with lower alcohol intake, but this was not statistically significant (*p* = 0.07) and had no effect on psychological well-being or an overall healthy lifestyle. Participation in LFS surveillance correlated with a healthy diet (*p* = 0.04) but not with the other lifestyle factors (smoking *p* = 0.92, PA *p* = 0.11, alcohol *p* = 0.49).

## 4. Discussion

This is to our knowledge the first nationwide analysis of lifestyle factors and health behavior among *TP53* PV carriers and their relatives. We found that women with a *TP53* PV had healthier diets and smoked less; however, there is an untapped potential for life-style-derived cancer prevention habits among this LFS cohort, who estimated their own cancer risk to be very high.

We had assumed that being aware of a CPS diagnosis and therefore knowing about the increased lifetime cancer risk would encourage a healthier lifestyle. However, our analysis revealed only slightly reduced smoking habits and a healthier diet of affected participants in comparison to their relatives but not to the general population. Similar results of unchanged life-style habits despite being confronted with a severe, life-threatening diagnosis have been described for young cancer patients maintaining an unhealthy lifestyle in respect of obesity, diet, and exercise after their cancer diagnosis [21].

A Mediterranean diet is an indicator for a healthy diet. It has not yet been followed in our cohort, although it is known to reduce cancer risk and also the risk of other diseases [22]. It was even slightly less frequently applied by men, concordant with previous findings [23]. These findings go along with previous reports in Germany, that women tend to eat heathier, e.g., more fruits and vegetables, then men [24]. For individuals with a CPS such as *BRCA1/2* PV carriers, a beneficial effect of a Mediterranean diet was described with a modulation of early breast cancer (BC) penetrance among women aged 18–30 due to body weight reduction of at least 10 pounds [25,26].

Furthermore, the distribution of BMI in our cohort corresponds to the German population aged 40–45 years [27]. Most study participants had at least a pre-obese BMI, which is associated not only with a risk for cancer, but also numerous other diseases [28]. In the present study, we did not elicit whether participants were aware of the impact of their lifestyle and BMI on their risk of developing cancer. A healthy dietary approach with its inherent cancer prevention characteristics is yet underachieved in our cohort and should be the focus of LFS-specific health education and self-empowerment. Even guidelines could help the affected individuals. It can be assumed that the risk of developing cancer could be reduced in *TP53* PV carriers by motivating them to conscientiously follow a Mediterranean diet. Current preclinical and clinical studies are underway to focus on pharmacological risk reduction via metabolic stimulation [5,29].

The commonly known carcinogens [30] of smoking and alcohol intake do increase cancer in general but especially in combination with an underlying CPS such as *BRCA*1/2 PV carriers [31] and were not abandoned in our high-risk cohort. On the contrary, the proportion of smokers in our study was significantly higher than the national average of 22.8%. However, in accordance with nationwide data, our study showed more male than female smokers [32]. In respect to alcohol consumption, the study population meets the distribution of alcohol intake in the German general population [33] and results of previous studies, e.g., outlining a higher alcohol consumption among people with a higher income [34]. However, other findings regarding a higher BMI associated with higher alcohol consumption [35] could not be confirmed in our rather small cohort.

PA has a cancer preventive effect in respect to cancer relapse and onset. The recommendations of the PA Guidelines for Americans, which include 15–20 MET hours for adults [36] was followed by the majority of the participants. However, its potential as a primary and tertiary cancer prevention strategy for CPS and improved QoL addressing fatigue and treatment tolerance are not yet sufficiently put into practice [37]. E-Health interventions offer the possibility to increase and monitor PA as shown for BC leading to long-lasting beneficial aspects [38] and furthermore harbor options to guide cancer prevention beside PA only. Issues concerning alcohol, obesity, and PA were almost equally distributed among *TP53* PV carriers and their relatives, pointing out a general need of support and guidance to self-derived cancer prevention not only for the affected but also for their relatives. Several other studies have pointed out the importance of a general healthy lifestyle (diet and body weight, PA, alcohol consumption, smoking) for people with an inherited CPS such as Lynch syndrome [39]. Although such mechanisms have not been prospectively investigated in *TP53* PV carriers, these findings and our descriptions underscore the need for patients’ education as well as prospective studies for detailed life-style recommendations to exhaust the cancer protective possibilities. As in our study, an unhealthy lifestyle was shown to correlate with poorer adherence to the surveillance program in other diseases, e.g., in patients with cirrhosis [40]. In our cohort the participation in surveillance was in general high and similar to that described in our preliminary study [3]. The present results clearly show that there are groups among *TP53* PV carriers who should be in focus in terms of their adherence not only to the surveillance program but also to a healthy lifestyle. These groups need more support to improve their life expectancy and quality.

Additionally, concordant to our findings, previous studies have outlined that children from households with lower levels of education are less likely to follow a cancer preventive lifestyle such as Mediterranean diet [41] and more likely to become smokers [42]; therefore, they need special attention. Interestingly, the proportion of university graduates was slightly higher compared with the general German population (34%) [43] as was the income of our study population compared to the general population with an average of EUR 2044 in 2021 [44]. The proportion of those with statutory insurance was similar to the general population. All aspects emphasize a gender- and socioeconomic-specific education on cancer prevention [45].

Beyond physical well-being, psychological aspects such as self-efficacy are also important motivational factors for a cancer preventive behavior [46]. Regarding the SF-12 questionnaire, our results confirm previous studies, for example, in women with BC [47], which showed that cancer (predisposition) reduced psychological but not physical well-being. The picture in our cohort is similar to that in a large survey in Germany [48], with men reporting better physical (50.55 vs. 49.49) and mental well-being (51.14 vs. 48.94) than women. Whereas the physical well-being among our cohort was considerably lower than in the general population (43.7 vs. 50.0) [48], neither a healthy diet nor PA influenced psychological or physical well-being.

The self-estimation of cancer risk is described as a psychological burden and a relevant factor for health care decision making in individuals with a CPS [49,50]. Interestingly, both relatives and *TP53* PV carriers realistically estimate their own risk of developing cancer, but those affected underestimate the risk of the general population, while relatives clearly overestimate the general lifetime cancer risk in Germany of 42% for women and 49% for men [51]. It has already been shown that communication about cancer risk can influence the screening behavior of high-risk families [52]. It would be hoped that this could also influence the lifestyle of affected individuals with a CPS.

The fact that smoking relatives but not the smoking *TP53* PV carrier rate their risk of developing cancer higher suggests that the relatives are probably aware of their unhealthy behavior. *TP53* PV carrier may assume that their pre-existing, elevated cancer risk will not be further increased even by harmful behavior.

The nature of our study inherits the possibility of selection bias as known from previous studies in this field [53]. The fact, that most participants were recruited through the consultation for hereditary breast and ovarian cancer may lead also to a selection bias Additionally, *TP53* PV carriers were able to select participating relatives among all their relatives, leading to an additional selection bias. As described previously, some affected individuals did not want their relatives to be interviewed, causing a lower participating rate [54]. However, the LFS relatives have yet rarely been studied [53] and this study was conducted in a diverse cohort of *TP53* PV carriers and their relatives.

## 5. Conclusions

Cancer prevention is a major goal of current nationwide campaigns such as the “National Decade Against Cancer” of the Federal Ministry of Education and Research in Germany. Especially in a population at high cancer risk, such as *TP53* PV carriers, it is useful to focus on patient and family education, particularly from childhood onward, especially for people with a lower level of education, to ensure a healthy and cancer-preventive behavior. To empower cancer prevention in this high-risk population, *TP53* PV carriers and their relatives should be made aware of possibilities to improve their lifestyle including on the one hand a reduction in noxious substances such as alcohol and cigarettes, and on the other hand preventive agents such as PA and a Mediterranean diet. Men in particular should be sensitized to the importance of a healthy diet and smoking cessation and women to the importance of integrating physical activity into everyday life. Adopting a healthy lifestyle needs support for concrete implementation in daily life by using, e.g., modern technologies such as apps. Larger prospective cohort studies for *TP53* PV carriers along with the recommended implementations are desirable for integrating cancer prevention into a treatment plan at the time of genetic diagnosis.

## Figures and Tables

**Table 1:** Characteristics of all study participants with a pathogenic/likely pathogenic (P/LP) *TP53* germline variant as well as their relatives as captured with the questionnaire.

	P/LP TP53 Variant Carriers (*n* = 70)	Relatives (*n* = 43)	*p*-Value
**Sex**			<0.01
Male *n* (%)	12 (17.14)	20 (46.51)	
Female *n* (%)	58 (82.86)	23 (53.49)	
**Age (years)**			0.27
Mean ± SD [range]	41.52 ± 12.11 [20–54]	38 ± 8.73 [22–66]	
Median	38	36	
**Years since P/LP *TP53* variant Diagnosis**			
Mean ± SD [range]	3.43 ± 3.28 [0–16]	4.95 ± 5.99 [0–25]	
Median	2.0	3.0	
0 (%)	3 (4)	1 (2)	
>10 (%)	2 (3)	3 (7)	
**Marital Status**			0.21
Single (%)	14 (20)	5 (12)	
In a relationship (%)	11 (16)	8 (19)	
Married (%)	41 (59)	24 (56)	
Divorced (%)	3 (4)	2 (5)	
Widowed (%)	1 (1)	4 (9)	
**Insurance Status**			1
State (%)	65 (93)	40 (93)	
Private (%)	5 (7)	3 (7)	
Highest School-Leaving Qualification			0.62
High school (%)	24 (35)	15 (35)	
College (%)	16 (23)	9 (21)	
University (%)	24 (35)	18 (42)	
Other (%)	5 (7)	1 (2)	
**Monthly Net Household Income (EUR )**			0.16
Mean ± SD [range]	3270 ± 2103 [600–9167]	3550 ± 1749 [450–7500]	
Median	2545	3200	
**Current Occupation**			0.56
Scholar/Student (%)	6 (9)	4 (9)	
Freelancer (%)	2 (3)	4 (9)	
Housewife (%)	3 (7)	2 (5)	
Employee (%)	42 (60)	24 (56)	
Public servant (%)	6 (9)	6 (14)	
Retired (%)	6 (9)	1 (2)	
Other (%)	5 (7)	2 (5)	
**Family (Question 1.9, 1.11, 1.12 ^1^)**			
Having children (%)	39 (56)	29 (67)	0.3
Child with cancer (%)	9 (13)	10 (23)	0.45
Child deceased due to cancer (%)	1 (1)	3 (7)	0.19
Sibling with P/LP TP53 variant (%)	25 (36)	1 (2)	<0.001
Sibling deceased due to cancer (%)	4 (6)	0 (0)	n.a. +
Parent deceased due to cancer (%)	13 (30)	1 (2)	0.07
**Own Cancer History (%)**	47 (67)	4 (9)	<0.001
**Cancer Risk Estimation (%)**			
Own mean ± SD [range]	71.78 ± 22.74 [15–100]	37.71 ± 21.27 [5–100]	<0.001
General mean ± SD [range]	37.89 ± 18.42 [1–90]	69.93 ± 29.88 [0–100]	<0.001
**Adherence to Surveillance Programm**			0.474
Yes, totally (%)	37 (53)	27 (63)	
Yes, partially (%)	24 (34)	12 (28)	
Not anymore (%)	0 (0)	1 (2)	
Never have (%)	4 (6)	1 (2)	
Do not know (%)	2 (3)	1 (2)	

Numbers are indicated with ± standard deviation and [range]; percentage is shown in (). SD = Standard Deviation, BMI = Body Mass Index, n.a. = not applicable; ^1^ questionnaire in the supplement; + no deceased siblings of relatives.

**Table 2:** Lifestyle and Dietary Habits, Physical Activity of people with a pathogenic/ likely pathogenic (P/LP) TP53 germline variant as well as their relatives as captured with the questionnaire.

	P/LP TP53 Variant Carriers (*n* = 70)	Relatives (*n* = 43)	*p*-Value
**BMI (kg/m²)**			0.66
Mean ± SD [range]	26.82 ± 7.31 [18.52–66.21]	25.83 ± 5.17 [18.22–40.72]	
Median	24.97	24.44	
**BMI Distribution (%)**			
<25 kg/m²	51	52	
25–<30 kg/m²	29	31	
30–<35 kg/m²	12	10	
35–<40 kg/m²	6	2	
>40 kg/m²	3	5	
**MEDAS score**	6.59	5.51	0.01
**Pack years**	1.93	4.51	0.14
**Physical Activity (metabolic equivalents)**	22.63	18.99	0.28

## Data Availability

The data that support the findings of this study are available from the corresponding author upon reasonable request.

## Supplementary Materials

The following supporting information can be downloaded at: https://www.mdpi.com/article/10.3390/curroncol29100614/s1, File S1: Translated self-designed questionnaire.
