# Supplementary material for: Health Behavior and Cancer Prevention among Adults with Li-Fraumeni Syndrome and Relatives in Germany—A Cohort Description
Source: Curr Oncol. 2022 Oct 15;29(10):7768–78. doi: 10.3390/curroncol29100614 (PMC9600238; doi:10.3390/curroncol29100614)
Supplement: Supplementary file 1 [file curroncol-29-00614-s001.zip › curroncol-1926625-supplementary.pdf]

Translated self-designed questionnaire

1. Demographic Data

1. When did you/your relative receive the Li-Fraumeni-Syndrome diagnosis? Please enter quarter and year.
2. Your date of birth:
3. You are:
   Female
   Male
   Divers
4. Your marital status:
   Single
   Single Parent
   Living in a partnership
   Married
   Divorced/separated
   Widowed
5. Highest school-leaving qualification
   None
   Special school certificate
   Lower secondary
   High School
   College
   University
   Other
6. How much do you weigh?
7. How tall are you?
8. Your current occupation:
   Scholar/Student
   Unemployed/Seeking a job
   Freelancer
   Housewife
   Employee
   Public Servant
   Pensioner
   Other
9. Do you have children?
   No
   Yes: Please enter Age and Sex
   Child 1
   Child 2
   Child 3
   Child 4
   Child 5
10. Have you ever had cancer in your life?
   Yes

No

11. Did one of your children have cancer?

Yes

No

12. Have you lost a child to cancer?

Yes

No

13. What kind of health insurance do you have?

State

Private

14. What is your average monthly net household income? (€)

15. Do you smoke?

Yes

No

Previously, but not for _______ years

16. How long have you smoked? How long did you smoke before? (years)

17. How much do (did) you smoke (before)? (Cigarettes per day)

18. How often do you drink alcohol?

Never

Once a month

2-4 times a month

2-3 times per week

4 or more times per week

19. If you drink alcohol in a day, how many alcoholic beverages do you typically drink? One glass of alcohol corresponds to: 0.33 L beer 0.25 L wine or sparkling wine 0.02 L spirits

I never drink alcohol

1-2

3-4

5-6

7-8

10 or more

2. Surveillance Program

Do you/Does your relative regularly participate in the recommended health and early detection examinations?

Yes, as recommended

Yes, but not all of them

Yes, but less often

No, not anymore

No, never

Don’t know

How high do you estimate your personal risk of developing cancer? ________ %

For relatives only: How high do you estimate the risk of your relative(s) getting cancer (again)? __________ %

How high do you estimate the risk of developing cancer in the average population?
__________ %
